# Equol an isoflavonoid: potential for improved prostate health, *in vitro *and *in vivo *evidence

**DOI:** 10.1186/1477-7827-9-4

**Published:** 2011-01-13

**Authors:** Trent D Lund, Crystal Blake, Lihong Bu, Amy N Hamaker, Edwin D Lephart

**Affiliations:** 1 Stoelting Co., Wood Dale, IL 60191, USA; 2 The Department of Physiology and Developmental Biology and the Neuroscience Center, Brigham Young University, Provo, Utah 84602, USA; 3 MRDDRC Imaging Core, Department of Neurobiology, Children's Hospital Boston and Harvard Medical School, Boston, MA 02115, USA

## Abstract

**Background:**

To determine: *in vitro *binding affinity of equol for 5alpha-dihydrotestosterone (5alpha-DHT), *in vitro *effects of equol treatment in human prostate cancer (LNCap) cells, and *in vivo *effects of equol on rat prostate weight and circulating levels of sex steroid hormones.

**Methods:**

First, *in vitro *equol binding affinity for 5alpha-DHT was determined using 14C5alpha-DHT combined with cold 5alpha-DHT (3.0 nM in all samples). These steroids were incubated with increasing concentrations of equol (0-2,000 nM) and analyzed by Sephadex LH-20 column chromatography. 14C5alpha-DHT peak/profiles were determined by scintillation counting of column fractions. Using the 14C5alpha-DHT peak (0 nM equol) as a reference standard, a binding curve was generated by quantifying shifts in the 14C5alpha-DHT peaks as equol concentrations increased. Second, equol's *in vitro *effects on LNCap cells were determined by culturing cells (48 hours) in the presence of increasing concentrations of dimethyl sulfoxide (DMSO) (vehicle-control), 5alpha-DHT, equol or 5alpha-DHT+equol. Following culture, prostate specific antigen (PSA) levels were quantified via ELISA. Finally, the *in vivo *effects of equol were tested in sixteen male Long-Evans rats fed a low isoflavone diet. From 190-215 days, animals received 0.1cc s.c. injections of either DMSO-control vehicle (n = 8) or 1.0 mg/kg (body weight) of equol (in DMSO) (n = 8). At 215 days, body and prostate weights were recorded, trunk blood was collected and serum assayed for luteinizing hormone (LH), 5alpha-DHT, testosterone and 17beta-estradiol levels.

**Results:**

Maximum and half maximal equol binding to 5alpha-DHT occurred at approximately 100 nM and 4.8 nM respectively. LNCap cells cultured in the presence of 5alpha-DHT significantly increased PSA levels. However, in the presence of 5alpha-DHT+equol, equol blocked the significant increases in PSA levels from LNCap cells. *In vivo *equol treatment significantly decreased rat prostate weights and serum 5alpha-DHT levels but did not alter LH, testosterone, and estradiol levels.

**Conclusions:**

Equol administration appears to have potential beneficial effects for prostate health and other 5alpha-DHT mediated disorders. Equol administration: reduces PSA levels from LNCap cells under 5alpha-DHT stimulation, decreases rat prostate size, decreases serum 5alpha-DHT levels and androgen hormone action, while not altering other circulating sex steroids or LH levels.

## Background

Polyphenols are a group of chemical substances found in plants that include berries, grapes, walnuts, peanuts, pomegranates, and other fruits and vegetables [1]. Many of these polyphenol preparations are available as dietary supplements [2]. The largest and best studied polyphenols are the dietary estrogen-like molecules or phytoestrogens [1]. Of the three main classifications of phytoestrogens: 1) isoflavones (derived principally from soybeans), 2) lignans (found in flaxseed in large quantities), and 3) coumestans (derived from sprouting plants like alfalfa), human consumption of isoflavones has the largest impact due to its availability and variety in food products. Isoflavones have been implicated as potential treatments for many disorders including cardiovascular disease, osteoporosis, age-related diseases, and hormone-dependent cancers [3,4].

However, these isoflavone molecules do not exist at high levels in their biologically active form in natural food products, but rather are at high abundance in a precursor form [3-5]. For example, daidzin, the precursor of daidzein, is the glycosidic form that contains a carbohydrate portion of the molecule. Daidzin is metabolized in the gastrointestinal tract by intestinal bacteria, which hydrolyze the carbohydrate moiety, to the biologically active isoflavone, daidzein [4,5]. Daidzein is then further metabolized in the intestine to equol at relatively low or high levels dependent upon several biological, dietary and presumably environmental factors [4,5]. Although, recent evidence suggests that equol is found naturally in white cabbage [6].

**Figure 1:**
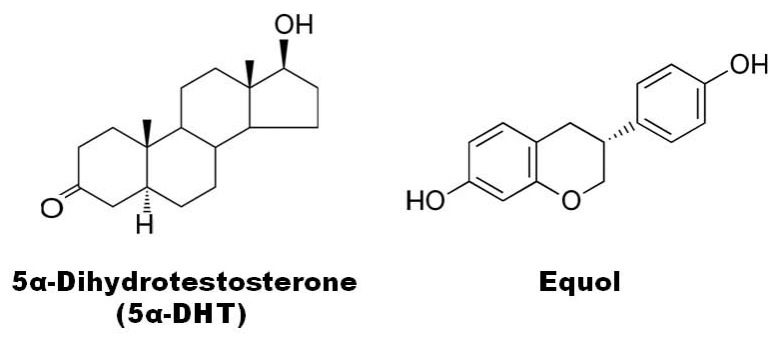
**Biochemical structures of dihydrotestosterone and equol**.

Equol has recently caught the interest of many researchers due to its rich antioxidant activity and implications in cancer research [4,5]. The chemical structure of equol contains a stereocenter at carbon number 3 which gives it two possible enantiomers and it has since been proven that the production of equol by microflora in mammals or other animals is selective for the S - enantiomer only [5]. S-equol has unique chemical properties compared to its R - enantiomer. S-equol has been shown to have a modest affinity for binding to and mimicking estrogen's effects on estrogen beta receptors (ERβ) due to its similar structure to natural estrogens [5,7]. However, S-equol shows little affinity for estrogen alpha receptors (ERα). Furthermore, equol (i.e., the R- and/or S-isomer) can act as an anti-androgen [7]. Equol's anti-androgen activity is unique as equol does not bind the androgen receptor (AR) but specifically binds 5α-dihydrotestosterone (5α-DHT) with high affinity, and thereby prevents DHT from binding the AR [7], see Figure 1. This finding is reconfirmed and extended here. Additionally, equol's mechanism of action, namely, its ability to specifically bind 5α-DHT and prevent 5α-DHT's biological actions in physiological processes, was studied.

For example, it is known that prostate cancer cells are supported in their growth by androgen stimulation and the androgen-regulated expression of the prostate specific antigen (PSA) is a biological marker of such stimulation [8]. Logically, any treatment that could decrease PSA levels in prostate cancer cells, or antagonize specific androgen hormone action, would have great potential in addressing prostate disorders, such as benign prostatic hyperplasia (BPH) or prostate cancer. With this in mind, we sought, in the following experiments, to determine equol's effect on 5α-DHT levels in cultured human prostate cancer (LNCap) cells. We also examined equol's effect on both prostate weight and circulating hormone levels *in vivo *using Long-Evans rats. In brief, the present results demonstrate that equol: a) (R- and/or S-isomeric mixtures) has high binding affinity for 5α-DHT making it a potent selective androgen modulator (SAM), b) blocks the stimulatory androgen action of 5α-DHT in increasing prostate specific antigen (PSA) levels in human cancer (LNCap) cell cultures and, c) significantly decreases serum 5α-DHT and subsequently prostate weight without altering, testosterone, 17β-estradiol or LH levels. Applications for equol to improve prostate disorders and other androgen-mediated conditions is also discussed.

## Methods

### Experiment 1. *In vitro *binding of Equol (Isomer Ratio) to 5α-DHT

#### Samples

In a 20 ml amber glass vial approximately 4,000 to 5,000 dpm of ^14^C5α- Dihydrotestosterone (DHT; Dupont/NEN, Boston, MA, USA) was added. Additionally, 3.0 nM (or approximately 865 pg/ml) of cold 5α-DHT (Sigma/Aldrich Chemical Co., St. Louis, MO, USA) was added (in all experiments) and where appropriate varying concentrations of equol (70% R-equol and 30% S-equol mixture; from 0 to 2,000 nM; obtained from LC Labs., Woburn, MA,USA or Robert Handa's Laboratory, Colorado State University, USA, R-equol or S-equol isomers only) from ethanol stock solutions. This mixture was vortexed for 30 seconds at room temperature and then dried down. Subsequently 1 ml of TEGMD (10 mM Tris CL, 1.5 mM EDTA, 10% glycerol, 25 mM molybdate and 1 mM dithrothreitol) buffer was added, capped, and mixed by inverting the vial 3-times. This mixture was incubated at room temperature for 20-24 hours before being placed on a Sephadex column [7].

#### Columns

Sephadex LH-20 (25-100 μm particle diameter) from Sigma/Aldrich Chemical Co. (St. Louis, MO, USA), was used to prepare 1.3 cm × 25 cm columns, as described by Lund et al [7]. Sephadex (0.25 grams) was mixed with 1.5 ml of TEGMD buffer. The serological pipette was plugged with a glass bead and 1 ml of Sephadex was loaded onto the column with TEGMD buffer and equilibrated. Flow rates under gravity were approximately 0.5 ml/min and 0.5 ml fractions were collected in plastic scintillation vials.

#### Quantification

Five ml of Ultima Gold scintillation fluid (Packard Instr. Co., Meriden, CT, USA) was added to each vial, mixed thoroughly and counted for 5 min. in a Beckmann LS 6500 scintillation counter. The peak ^14^C5α-DHT fraction and profile using 0 nM of equol was used as reference. The shift (advancement) in peak/profile in subsequent experiments using increasing concentrations of equol (70% R-isomer and 30% S-isomer in this experiment) was calculated based upon this reference (as arbitrary binding units as a percentage of the original reference, n = 6 for 0 nM of equol). Note: data not shown, 2,000 nM of equol was tested in this experiment which yielded 100.0% ± 0.3 (s.e.m.) binding, n = 3. Finally, over thirty steroid hormones were tested (see Table 1) (in preliminary studies to determine the selectivity and specificity of equol for binding 5α-DHT). The steroids were purchased from Steraloids, Inc., Newport, RI, USA or Sigma/Aldrich Chem. Co., St. Louis, MO, USA.

**Table 1:** Steroid compounds tested in Equol Binding Assays

**Chemical Name**	**Trivial Name**
**4-ANDROSTEN-17β-OL-3-ONE**	**TESTOSTERONE**
**5-ANDROSTEN-3β-OL-17-ONE**	**DHEA**
**5α-ANDROSTAN-3α,17β-DIOL**	**17β-DIHYDROANDROSTERONE**
**5α-ANDROSTAN-3β,17α-DIOL**	**NA**
**5α-ANDROSTAN-3β,17β-DIOL**	**NA**
**5α-ANDROSTAN-3,17-DIONE**	**ANDROSTANEDIONE**
**5α-ANDROSTAN-17β-OL-3-ONE**	**5α-DHT**
**5β-ANDROSTAN-17β-OL**	**NA**
**5β-ANDROSTAN-17β-OL-3-ONE**	**5β-DHT**
**4-ANDROSTEN-3,17-DIONE**	**ANDROSTENEDIONE**
**1,3,5(10)-ESTRATRIEN-3,17α-DIOL**	**EPIESTRADIOL**
**1,3,5(10)-ESTRATRIEN-3,17β-DIOL**	**ESTRADIOL (E2)**
**1,3,5(10)-ESTRATRIEN-3,16α,17β-TRIOL**	**ESTRIOL (E3)**
**1,3,5(10)-ESTRATRIEN-3-OL-17-ONE**	**ESTRONE (E1)**
**5α-ESTRAN-3,17-DIONE**	**5α-DIHYDROANDROSTENEDIONE**
**5-PREGNEN-3β-OL-20-ONE**	**PREGNENOLONE**
**4-PREGENEN-3,20-DIONE**	**PROGESTERONE (P4)**
**5α-PREGNAN-3α-OL-20-ONE**	**ALLOPREGNANOLONE**
**5α-PREGNAN-11β,21-DIOL-3, 20-DIONE**	**ALLODIHYDROCOSTERONE**
**5α-PREGNAN-3α,11β,21-TRIOL-20-ONE**	**ALLOTETRAHYDROCORTICOSTERONE**
**5α-PREGNAN-3β, 21β, 21-TRIOL-20-ONE**	**EPIALLOTETRAHYDROCORTICOSTERONE**
**5α-PREGNAN-3β,11β,17,21-TETROL-20-ONE**	**3β, 5α-TETRAHYDROCORTISOL**
**5α-PREGNAN-11β,17,21-TRIOL-3,20-DIONE**	**ALLODIHYDROCORTISOL**
**5α-PREGNAN-17,21-DIOL-3,11,20-TRIONE**	**ALLODIHYDROCORTISONE**
**5α-PREGNAN-3β-OL-20-ONE**	**5α-DIHYDROPREGNANOLONE**
**5α-PREGNAN-3, 20-DIONE**	**5α-DIHYDROPROGESTERONE (5α-DHP)**
**5β-PREGNAN-3α-OL-20-ONE**	**3α-HYDROXY-5βTETRAHYDROPROGESTONE**
**5β-PREGNAN-11β,21-DIOL-3,20-DIONE**	**5β -DIHYDROCORTICOSTERONE**
**5β-PREGNAN-3α,11β,21-TRIOL-20-ONE**	**TETRAHYDROCORTICOSTERONE**
**5β-PREGNAN-3α,11β,17,21-TETROL-20-ONE**	**TETRAHYDROHYDROCORTISOL**
**5β-PREGNAN-11β,17,21-TRIOL-3,20-DIONE**	**5β-DIHYDROCORTISOL**
**5β-PREGNAN-17,21-DIOL-3,11,20-TRIONE**	**5β -DIHYDROCORTISONE**
**5β-PREGNAN-3,20-DIONE**	**5β-DIHYDROPROGESTERONE (5β-DHP)**

### Experiment 2. *In vitro *blockade of 5α-DHT stimulated Prostate Specific Antigen (PSA) levels in LNCap cells by equol: Human Prostate Cancer Cell Cultures

Human prostate cancer cell line was obtained from American Type Culture Collection (ATCC; Manassas, VA, USA; ATCC # CRL-1740, LNCap-FGC) and cultured in a 37° C, humidified incubator with 5% CO_2_, in RPMI Medium (Sigma/Aldrich Cat. # R-8758) with 5% fetal bovine serum (Hyclone/Thermo Sci., Waltham, MA, USA, Cat. # SH30088.03, Lot number APC20780) and 5 mM Hepes (Sigma/Aldrich Cat. H-0887), 1× antibiotic/antimycotic (Sigma/Aldrich Cat. # A5955). Cells were expanded in T-150 flasks for three passages until cryopreservation and storage in liquid nitrogen in RPMI medium with 10% FBS and 10% DMSO as cryopreservative. A cryovial was then thawed in a 37° C water bath, expanded again one or two passages, and then plated at 10,000 cells per 96 well in 0.2 ml medium in 96 well plates in RPMI 5% FBS medium. After approximately 48 hours, the medium was changed to phenol red-free DMEM/F12 (Gibco/Invitrogen, Carlsbad, CA, USA, Cat. #21041-025) with 2% FBS and 1× antibiotic/antimycotic and test materials (5α-DHT, equol or 5α-DHT plus equol; n = 6 per treatment) and DMSO/vehicle controls were added at the appropriate concentration (either, 0.1 nM, 1.0 nM or 10 nM of 5α-DHT or 100 nM, 10 nM or 1.0 nM of equol) from 10× stocks for specific experiments. [The change to phenol red-free medium was to eliminate any potential estrogenic influence]. Cells were cultured for approximately 48 hours in the presence of the test materials and controls prior to removal of medium supernatants for prostate specific antigen (PSA) ELISA quantification.

#### MTT Assay

Cytotoxicity was determined by spectrophotometric detection of reduced 3-(4,5-dimethylthiazol-2-yl)-2,5-diphenyltetrazolium bromide (MTT, Sigma/Aldrich Cat # 5655) at 550 nm using a Molecular Devices Vmax 96 well plate reader and SoftMax software (n = 6 per treatments or controls). Metabolic activity can be used as a measure of cytotoxicity, in that the intensity of the reduced form of MTT by live cells is directly proportional to cellular viability, and inversely proportional to cytotoxicity.

#### Cellular DNA Assay

Cellular DNA was determined using a CyQuant Cell Proliferation Kit (Molecular Probes/Invitrogen, Carlsbad, CA, USA, Cat # C7026) according to the manufacture's instructions to quantify cellular proliferation.

#### PSA ELISA

Tissue culture supernatants were diluted 10-fold in PBS and stored at -20°C, then thawed at room temperature prior to assay. A commercial ELISA kit for free PSA (Bio-Quant, San Diego, CA, USA, Cat. # BQ 067T) was utilized according to the manufacturer's instructions, and data was determined using a Molecular Devices Vmax 96 well plate reader and SoftMax software.

### Experiment 3. *In vivo *blockade of the stimulatory effects of 5α-DHT by equol

This is an example of equol preventing the stimulatory effects of 5α-DHT *in vivo*. Rats were injected with 1 mg of non-racemic equol (52% S-isomer, 48% R-isomer) for 25 consecutive days, and serum 5α-DHT levels and prostate weights were measured. Adult (50 day-old) males (n = 16), purchased from Charles River Laboratories (CRL; Wilmington, MA, USA), were caged individually and housed in the Brigham Young University Vivarium and maintained on an 11-dark, 13-hour light schedule (lights on 0600-1900). Before purchase, the male animals were fed a diet containing approximately 200 ppm of isoflavones at the supplier (CRL). At 50 days of age, upon arrival, the male rats were placed on a diet containing approximately 10 ppm of isoflavones; referred to hereafter as the low isoflavone diet (Zeigler Bros., Gardnes, PA, USA; Phytoestrogen Reduced Rodent Diet II). All animals remained on the low isoflavone diet until 216 days of age to exclude the influence of dietary isoflavones on the measured parameters. At 150 days of age the rats were divided into two groups (control or equol treatments) that were matched by age and body weight. Starting at 190 days of age the male rats received a daily subcutaneous 0.1cc injection at the nape of the neck of vehicle (n = 8) (dimethyl sulfoxide; DMSO) or equol (n = 8) at a dose of approximately 1.0 mg/kg for 25 consecutive days.

The body weights for each group were recorded weekly starting at 150 days of age before the treatments were initiated, with weights obtained immediately before and after the treatments were administered (there were no significant differences in body weights between the control and equol groups at the start of this experiment). At 216 days of age the animals were weighed [grams (g) ± 0.1 g], then anesthestized with Ketamine/acepromazine and blood was collected from the heart. Next the ventral prostate organ was dissected and weighed [milligrams (mg) ± 0.001 mg]. The collected blood samples were centrifuged and serum was stored at -20˚ C until assayed. All collection procedures were performed blind to the treatments. This animal protocol was approved by the Institutional Animal Care and Use Committee at Brigham Young University.

Serum testosterone, 5α-DHT, and 17β-estradiol were quantified by radioimmunoassay (RIA) kits purchased from Diagnostic System Laboratories (Webster, TX, USA). Luteininzing hormone (LH) was quantified by an assay utilizing standards from the National Institutes of Health (NIH) USA pituitary hormone program. The samples were run in duplicate for each RIA, with internal control samples. In all RIAs, the control values were within normal ranges. The intra-assay coefficients of variance for the assays were: testosterone = 6.0%; for 5α-dihydrotestosterone = 8%, 17β-estradiol = 5% and LH = 9%.

#### Reagents and supplies

All reagents and supplies not specifically mentioned (e.g., EDTA, DMSO, glycerol, dithrothreitol, pipettes, 1.3 × 25 cm columns, etc.) were purchased from Sigma/Aldrich Chem. Co., St. Louis, MO, USA.

#### Statistical analyses

Where appropriate, data were analyzed by analysis of variance statistics (ANOVA) followed by Newman-Keuls post hoc tests. Significance was p < 0.05. Curve fitting, scientific graphing, and analysis were completed using GraphPad Software (GraphPad Prism 3.0, San Diego, CA, USA).

## Results

### Experiment 1

In preliminary studies, Table 1 shows 33 different steroid compounds that were tested in binding assays (outlined above) to determine equol's affinity for binding to each. While equol has modest affinity for some 5α-reduced steroids, equol displayed the highest affinity for 5α-dihydrotestosterone (5α-DHT) and had no affinity for 5β-dihydrotestosterone (5β-DHT) or some of the most common natural sex steroids, such as: estradiol, estrone, estriol, progesterone or testosterone. [Notably: as tested in these assays, the isoflavones: genistein and daidzein do not bind 5α-DHT].

**Figure 2:**
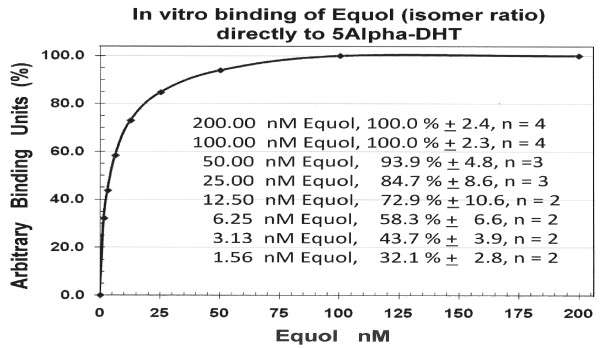
**In vitro binding of equol (isomer ratio) to 5α-DHT**. ^14^C5α- Dihydrotestosterone served as the tracer and 3.0 nM (or approximately 865 pg/ml) of cold 5α-DHT was added (in all experiments) and where appropriate varying concentrations of equol (70% R-equol and 30% S-equol mixture; from 0 to 2,000 (see methods). All data expressed as the mean of the percent binding ± SEM (see insert).

As shown in Figure 2, using ^14^C5α-DHT as the tracer in the presence of 3.0 nM (or approximately 865 pg/ml) of cold DHT (in all samples, representing a high-normal level of circulating 5α-DHT in men) varying concentrations of equol (70% R-equol and 30% S-equol mixture) from 0 to 2,000 nM yielded the binding curve displayed in this Figure. [The 2,000 nM dose is not graphically displayed in this Figure]. Maximum and half maximal equol binding to 5α-DHT occurred at approximately 100 nM and 4.8 nM, respectively. The number of samples tested at each concentration of equol examined ranged between 2 to 6 replicates (see Figure 2). [For conversion purposes, a concentration of 10.3 nmol/L of equol is approximately equivalent to 2.5 ng/ml of equol].

Notably, equol at a 70% R-isomer and 30% S-isomer ratio was tested in this experiment. However, racemic equol or equol at a 30% R-isomer and 70% S-isomer ratio when tested in these assays yielded similar results to that displayed in Figure 2. These data suggest that equol, regardless of the isomer ratio mixture can binding selectively 5α-DHT *in vitro*.

### Experiment 2

**Figure 3:**
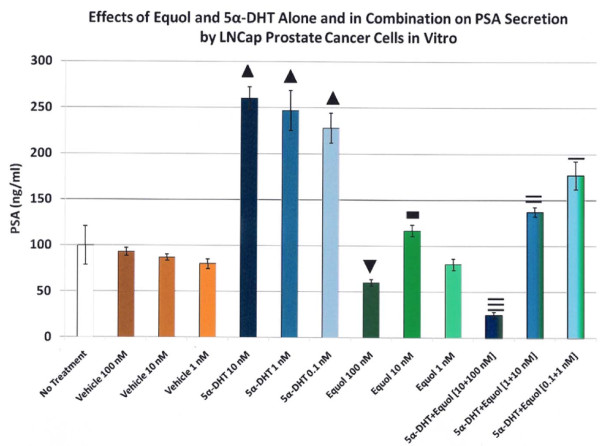
***In vitro *evidence: The effects of 5α-DHT or equol alone and in combination on prostate specific antigen (PSA) by LNCap Human Prostate Cancer Cells**. All data expressed as the mean + the standard error of the mean (sem). Up black triangle significantly greater levels in all 5α-DHT treatments compared to all other treatments (p < 0.0001) Down black triangle, significant decrease at 100 nM of equol vs. all control values (p < 0.0001). Black square, slight but significant increase in PSA levels in the equol 10 nM group compared to DMSO-vehicle values (p < 0.008) but not to the no-treatment group (p < 0.47). Three horizontal lines, significant decrease at 10 nm of 5α-DHT plus equol at 100 nM compared to 10 nM of 5α-DHT alone (p < 0.0001). Two horizontal lines, significant decrease at 1 nM of 5α-DHT plus equol at 10 nM compared to 1 nM of 5α-DHT alone (p < 0.001). One horizontal line, represent a significant decrease at 0.1 nM of 5α-DHT plus equol at 1 nM compared to 0.1 nM of 5α-DHT alone (p < 0.042).

This is an example of the effects of equol preventing the stimulatory effects of 5α-DHT in LNCap prostate cancer cells *in vitro *from secreting prostate-specific antigen (PSA), a molecule known to be regulated by 5α-DHT, as measured by PSA ELISA (Figure 3). Treatment with 0.1, 1 or 10 nM of 5α-DHT; 1, 10 or 100 nM of equol or a combination of 5α-DHT plus equol (0.1 nM 5α-DHT and 1 nM equol; 1 nM 5α-DHT and 10 nM equol or 10 nM 5α-DHT and 100 nM equol). Each obtained value represents 6 replicates + standard error of the mean (sem). Cytotoxicity, as assessed by MTT Assay, did not influence PSA production by LNCap prostate cancer cells for any of the treatments or controls (data not shown). Also, while DHT treatment has been reported to increase cellular proliferation of this cell line *in vitro *[9], none of the treatments significantly altered cellular proliferation as quantified by the cellular DNA assay (data not shown).

As shown in Figure 3: In the 1, 10 or 100 nM Vehicle (DMSO) treatments PSA levels did not differ from the No Treatment baseline group and controls were not significantly different from each other. Treatment with 0.1, 1 or 10 nM 5α-DHT stimulated PSA secretion to maximal levels (ranging from 228.3 to 260 ng/ml). PSA levels from cells treated with 1, 10 or 100 nM equol were well below DHT-treated values. In fact, the 100 nM equol treatment alone yielded a PSA level at 60 ng/ml that was significantly decreased below all vehicle-control values (at approximately 87 ng/ml). The equol treatment at 10 nM slightly but significantly increased PSA levels (to 116.7 ng/ml) compared to all the DMSO-vehicle-control values around 87 ng/ml, but not to the no-treatment group at 100 ng/ml). [This may be due to the number of replicates (6) in this experiment where standard error of the mean (sem) values were relatively small]. However, combinations of 10 nM 5α-DHT plus 100 nM equol, as well as 1 nM 5α-DHT plus 10 nM equol, and 0.1 nM 5α-DHT plus 1 nM equol treatments abrogated the increase in PSA secretion, compared to the 5α-DHT treatments alone, at the respective androgen concentrations (see Figure 3). Taken together with the *in vitro *binding demonstrated in Figure 2, these data from experiment 2, suggest that equol binds the 5α-DHT molecule and biologically inactivates it in human cancer prostate cells.

### Experiment 3

To determine whether equol can bind 5α-DHT *in vivo*, adult male Long-Evans rats were treated with equol for 25 consecutive days (@ 1 mg/kg via s.c. injections). Equol-injected animals displayed an approximately 50% decrease in serum 5α-DHT compared to (DMSO) vehicle-injected control animals (Table 2). This finding corresponds with the significant decrease in androgen hormone action which is known to regulate prostatic cell proliferation, and hence, prostate weight. Prostate weights were significantly decreased by approximately 20% in the equol-injected males compared to control rats (either alone or when prostate weights were standardized with body weights).

**Table 2:** Serum 5α-DHT levels, prostate weights and reproductive parameters in Equol-treated male rats

Parameter measured	Vehicle	Equol	Change
**Prostate Weight (PW), mg**	**535 ± 23**	429 ± 30**	**20%↓**
**PW/100 g Body Weight**	**76 ± 4**	61 ± 5**	**20%↓**
**Luteinizing Hormone (LH), ng/ml**	**1.6 ± 0.2**	**1.3 ± 0.1**	**NSC**
**Serum Testosterone, ng/ml**	**2.1 ± 0.4**	**2.3 ± 0.5**	**NSC**
**Serum 5α-DHT, pg/ml**	**100 ± 18**	52 ± 5*	**50%↓**
**Serum 17β-Estradiol, pg/ml**	**3.4 ± 0.6**	**4.8 ± 0.7**	**NSC**

n = 8 animals per group. NSC = no significant change. The data are expressed as the mean ± standard error of the mean (sem) for each parameter.

** = p < 0.016 * = p < 0.001

When LH and testosterone were quantified between the treatment groups there were no significant differences in these hormone levels (Table 2). Since LH is the gonadotrophin regulating testosterone synthesis from Leydig cells in the testes, this is not a surprising result. Finally, when 17β-estradiol levels were determined there were no significant differences between the treatment groups. All hormone levels were within normal ranges of that expected for this strain, age and sex of rat. Notably, Long-Evans rats do not develop spontaneously prostate cancer but enlargement of prostatic tissue is observed with aging.

This *in vivo *study demonstrates that equol can contact and biologically inactive the 5α-DHT molecule as shown by the significant decrease in 5α-DHT levels in blood and significantly reduced prostate weights of equol-treated male rats. Finally, the *in vitro *and *in vivo *studies reported above demonstrates that equol may be effective in treating prostate disorders or other androgen-mediated diseases that are regulated by the hormone 5α-DHT.

## Discussion

The binding assays in the present study confirm and extend previous findings from our laboratories [7]. However, this protein and cell-free assay represents a system that is absent of steroid-binding proteins and other biologicals that may influence the *in vivo *interaction between equol and 5α-DHT. Moreover, after testing over 30 steroid hormones with various androgenic, estrogenic or progestin chemical configurations, equol (racemic or non-racemic mixtures) specifically binds 5α-DHT with apparent equal affinity since the bind curves obtained were very similar using different equol isomer ratios. This suggests several important issues. First, all humans have plasma levels of S-equol circulating in their bloodstream that may represent a natural modulation of the potent androgen, 5α-DHT, regardless of whether or not they are "equol producers" [4,5]. Second, this circulating equol is derived from consumption of soy- or high content isoflavone-containing food products or low abundant isoflavone foods such as corn, wheat, cow's milk, etc., as a primary consumer. Finally, because grazing or other animals that produce high levels of S-equol naturally that are then consumed, such as meat products, by humans represents a source of equol as a secondary consumer. Evidence to support this last notion is shown in humans that consume high meat and fat diets display higher blood isoflavone/equol levels [10].

This denotes an interesting 'natural' modulation of 5α-DHT in humans, since all individuals consume food products that contain the precursor daidzein molecule or equol itself (i.e., soy, corn, wheat, cow's milk or animal meat products, etc.) [4,6,11-13]. In fact, whether or not swine were fed a soy-containing or protein-supplemented diet the serum equol levels between the dietary treatment groups were essential the same [14]. Since the apparent half-maximal binding of equol to 5α-DHT occurred at 4.8 nM (or approximately 1.2 ng/ml) the very low level of circulating equol may alter androgen hormone action in a manner previous unknown in normal physiological conditions. Also, the apparent half-life of R- or S-equol in humans is 5 to 6 hours [5], suggesting a viable time interval and mechanism of action. While the present data were derived from *in vitro *binding studies that does not represent the complex physiological environment this viewpoint will be covered below in reference to prostate health.

Further evidence presented in this study for equol binding 5α-DHT is presented in the experiment where equol blocked the stimulatory androgen action of 5α-DHT in increasing PSA levels in human cancer cell cultures. Although, this may be the major mechanism of action, it is known that equol (particularly S-equol) has an affinity for ERβ [5]. Previous laboratories, including ours, have shown that isoflavones binding to ERβ in the prostate can down regulate the androgen receptor (AR) and thus decrease androgen hormone action [15]. Since PSA is an androgen-regulated gene this idea may have merit [16,17]. In addition to equol directly binding 5α-DHT, it may alter AR expression and hence significantly decreased PSA levels in the present study. Support for this view comes from the significant decrease in PSA levels with equol treatment alone (@ 100 nM) in experiment 2 that displayed values even below baseline (vehicle) PSA concentrations. However, it could be argued that the fetal bovine serum provided a background androgenic stimulation and this may also account for this observation. In any event, the results of this experiment demonstrate the positive influence equol has on PSA levels and implicates potential applications of equol for prostate disorders. Finally, phase II clinical trial data examining dietary intervention with isoflavone supplementation in men with recurrent prostate cancer display a decline in PSA levels that support our present *in vitro *data [18].

As mentioned earlier, equol has the ability to bind ERβ. This may explain how isoflavones accumulate in prostate tissue and prostatic fluid after oral supplementation [19-21] and it has been shown that binding ERβ in the prostate decreases inflammation and carcinogenesis [22]. Furthermore, this not only applies to prostate health but to other tissue-specific sites in the body where ERβ is present. For example, there is an abundance of ERβ in the brain, specifically in the frontal cortex and raphe nuclei as well as other brain regions that may be involved in mechanisms of anxiety and depression [23-25]. Preliminary data from our laboratory suggest that equol treatment in aged-rats after natural ovarian failure is effective as an anti-depressive agent at low concentrations that significantly increase serotonin levels. Additionally, the skin represents the largest organ of the body where ERβ is abundant in the epidermal and dermal regions, especially the keratinocytes and fibroblasts, respectively which may be tissue sites for beneficial alterations in androgen hormone action [26]. For instance, it is known the androgens decrease, whereas, estrogenic compounds enhance wound healing [27] that may have effective applications for topical treatments.

The results of the third experiment demonstrated that equol can significantly decrease serum 5α-DHT along with a significant decline in prostate weight without altering, testosterone, 17β-estradiol or LH levels. There is evidence that Asian cultures have lower prostate cancer rates compared to Western cultures [1,3,28]. Furthermore, when data is stratified examining individuals that produce equol naturally after soy consumption suggest that lower cancer rates are due to the beneficial influence of this isoflavonoid molecule [29].

Moreover, while equol has been shown to have positive benefits in human prostate health, there is evidence that R-, rather than the S - enantiomer is responsible for the *in vivo *chemoprotective properties of equol [30].

Additionally, from pilot data, serum 5α-DHT levels significantly decreased by 10 to 15% in men (50 to 60 years of age) and 20 to 26% in postmenopausal women (60 to 65 years of age) when an equol oral dose of 3 mg per day was administered (without side effects), suggesting that equol effectively binds and/or alters 5α-DHT levels. This pilot data is supported by a recent study showing isoflavone supplementation that increased the production of serum equol in equol-producers resulted in a decline in serum 5α-DHT levels in men by approximately 18% versus before supplementation values [31].

## Conclusions

In summary, our *in vitro *studies reveal equol binds specifically 5α-DHT and prevents increases in PSA from LNCap cells, while the *in vivo *studies demonstrate that equol decreases rat prostate size, decreases serum 5α-DHT levels and androgen hormone action, while not altering other circulating sex steroids or LH levels. Taken together these data suggest a potential use of equol as a therapeutic agent for men's prostate health. For example, benign prostate hyperplasia (BPH or prostate enlargement) that is manifested by nocturia (need to urinate frequently during the sleep-cycle), frequency of urination during the day or night, decreased voided urine volume, sensory urgency to urinate, inability to start and stop a urine stream, etc., where equol may mediate androgen hormone action of 5α-DHT and/or androgen steroid receptor inactivation is influenced by binding ERβ in a positive manner.

## List of abbreviations used

(5α-DHT): 5α- dihydrotestosterone; (AR): androgen receptor; (BPH): benign prostate hyperplasia; (PSA): prostate specific antigen; (LNCap): lymph node metastatic lesion of human prostatic adenocarcinoma; (DMSO): dimethyl sulfoxide; (SAM): selective androgen modulator; (ERβ): estrogen receptor beta; (LH): luteinizing hormone; (ELISA): enzyme-linked immunosorbent assay.

## Competing interests

The authors declare that they have no competing interests.

## Authors' contributions

TDL designed and performed initial binding studies, analyzed data sets and authored portions of this paper. CB analyzed data sets and authored portions of this paper. LB conducted and performed the blood steroid/hormone assays in all the studies and performed portions of the *in vivo *animal studies, analyzed data sets and authored portions of this paper. ANH analyzed data sets and authored portions of this paper. EDL designed and performed the *in vitro *binding assays, LNCap studies and *in vivo *animal studies, analyzed data sets, authored the paper and obtained funding. All authors read and approved the final manuscript.

## Authors' information

Trent D. Lund, PhD, is president of Stoelting Co., Wood Dale, IL 60191, USA. Crystal Blake, PhD, is a research associate in Physiology and Developmental Biology at Brigham Young University (BYU), Provo, Utah 84602, USA. Lihong Bu, PhD, is MRDDRC Imaging Core manager and instructor, Department of Neurobiology, Children's Hospital Boston and Harvard Medical School, Boston, MA 02115, USA. Amy N Hamaker, is an undergraduate student majoring in Neuroscience at Brigham Young University, Provo, Utah 84602, USA. Edwin D. Lephart, is professor of Physiology and Developmental Biology and The Neuroscience Center at BYU, Provo, Utah, 84602, USA.
